# Supplementary material for: Single-cell analysis identifies genes facilitating rhizobium infection in Lotus japonicus
Source: Nat Commun. 2023 Nov 7;14:7171. doi: 10.1038/s41467-023-42911-1 (PMC10630511; doi:10.1038/s41467-023-42911-1)
Supplement: Supplementary file 1 — Supplementary Information [file 41467_2023_42911_MOESM1_ESM.pdf]

Supplementary Information

Single-cell analysis identifies genes facilitating rhizobium infection in *Lotus japonicus*

Manuel Frank[1,*], Lavinia Ioana Fechete[1,*], Francesca Tedeschi[1], Marcin Nadzieja[1], Malita Malou Malekzadeh Nørgaard[1], Jesus Montiel[1,2], Kasper Røjkjær Andersen[1], Mikkel H. Schierup[3], Dugald Reid[1,4] and Stig Uggerhøj Andersen[1]

*: These authors contributed equally to the work.

[1] Department of Molecular Biology and Genetics, Aarhus University, Universitetsbyen 81, DK-8000 Aarhus C, Denmark.

[2] Center for Genomic Sciences, National Autonomous University of Mexico. Cuernavaca, Mexico.

[3] Bioinformatics Research Centre, Aarhus University, Universitetsbyen 81, DK-8000 Aarhus C, Denmark.

[4] Department of Animal, Plant and Soil Sciences, School of Agriculture, Biomedicine and Environment, La Trobe University, Melbourne, Australia

Authors for correspondence: Dugald Reid (dugald.reid@latrobe.edu.au) and Stig U. Andersen (sua@mbg.au.dk).

Supplementary figures

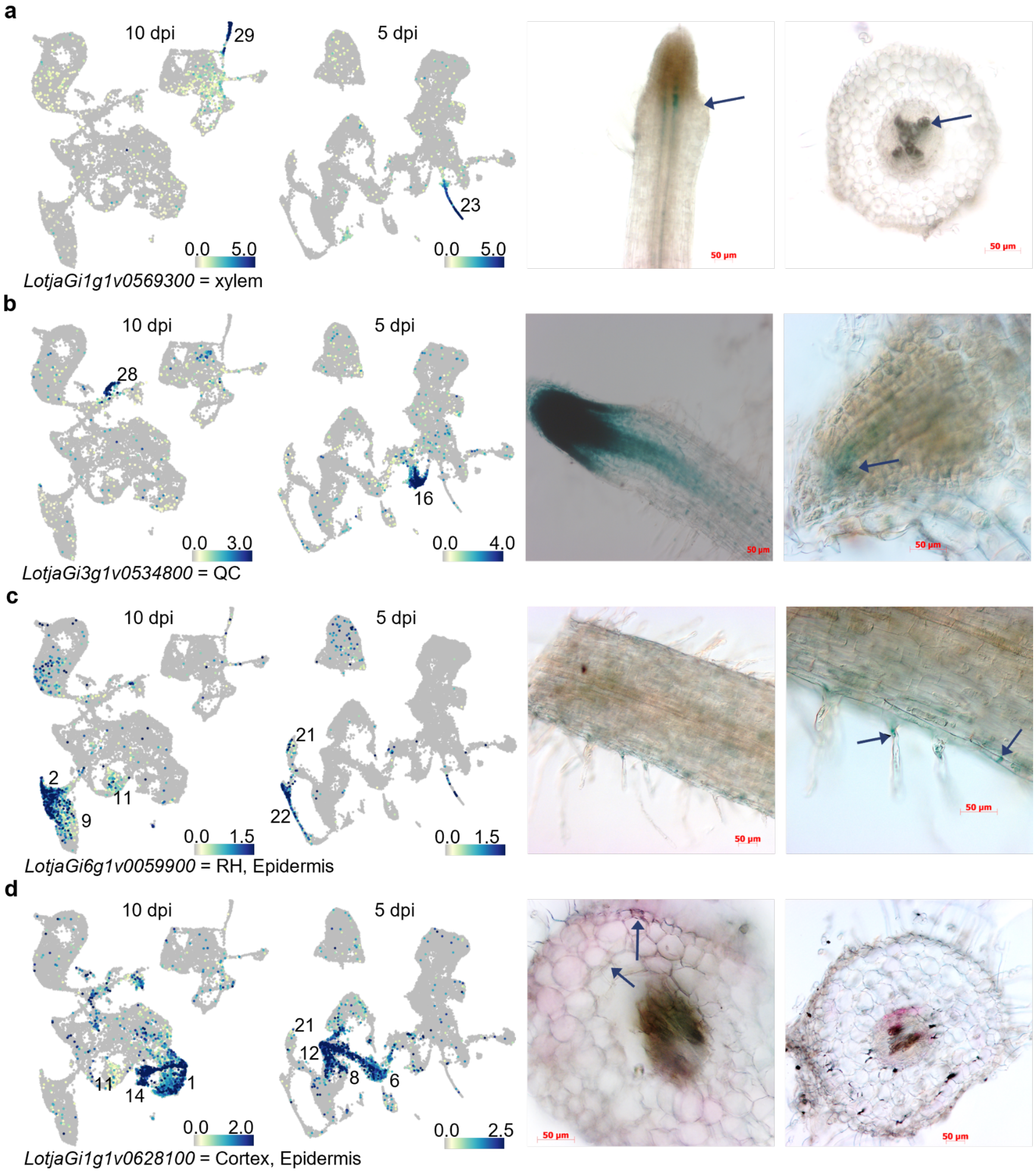

Supplementary figure 1. Validation of annotation of xylem, QC, root hair/epidermis and cortex/epidermis clusters. Normalized expression of (a) *LotjaGi1g1v0569300*, (b) *LotjaGi3g1v0534800*, (c) *LotjaGi6g1v0059900* and (d) *LotjaGi1g1v0628100* in the ten and five dpi data sets and images of respective *promoter:GUS/RUBY* expressing *Lotus* roots. Scale bar: 50 µm.

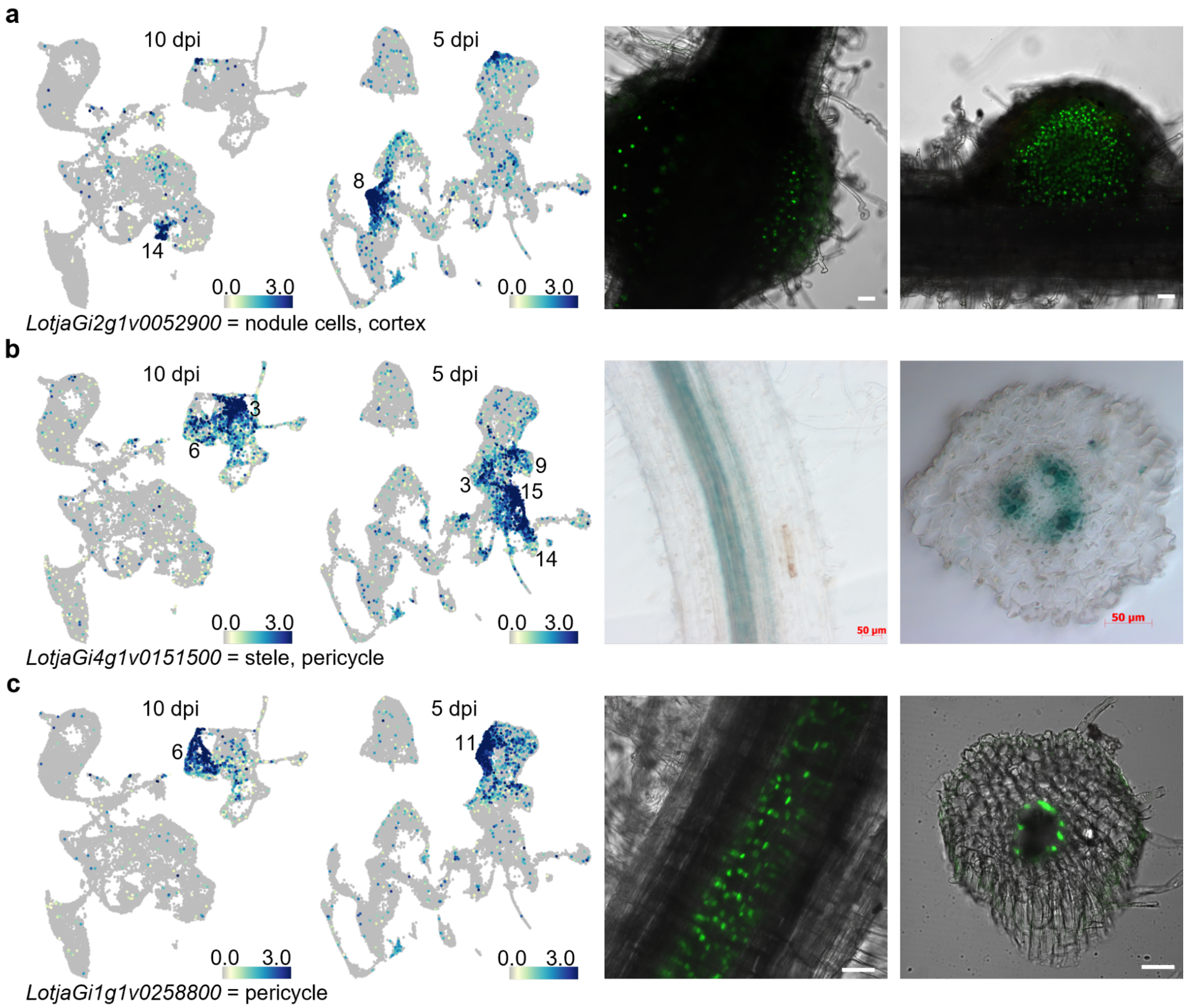

Supplementary figure 2. Validation of annotation of nodule cell, stele and pericycle cell clusters. Normalized expression of (a) *LotjaGi2g1v0052900*, (b) *LotjaGi4g1v0151500* and (c) *LotjaGi1g1v0258800* in the ten and five dpi data sets and images of respective *promoter:tYFPnls/GUS* expressing *Lotus* roots. Scale bar: 50 µm.

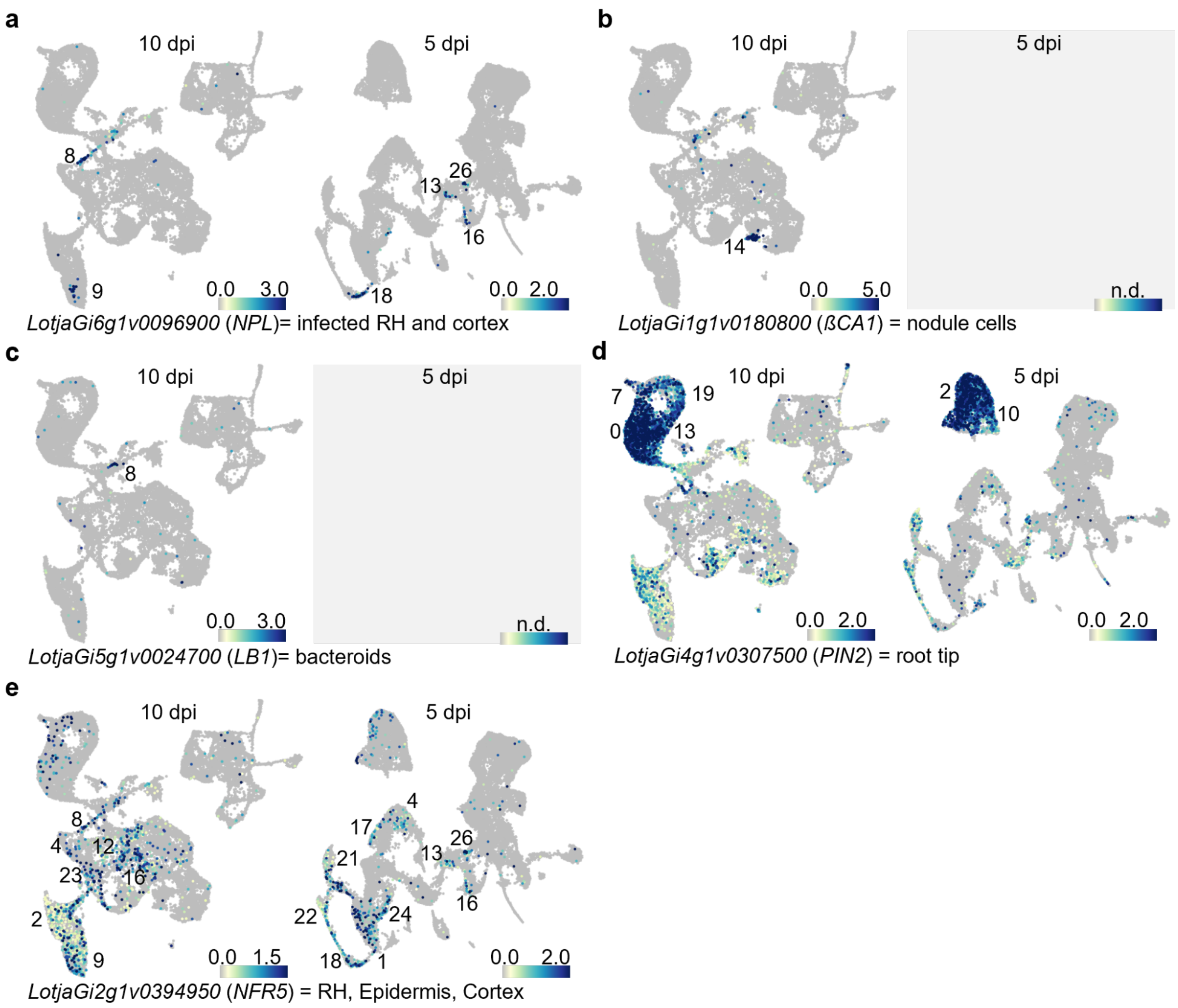

Supplementary figure 3. Validation of infected RH and cortex, nodule and bacteroid-containing cell, root tip, epidermis and cortex clusters. Normalized expression of previously published tissue marker genes (a) *NPL*[24], (b) *βCA1*[46], (c) *LB1*[47], (d) *PIN2*[66] and (e) *NFR5*[52] in the ten and five dpi data sets. Images of respective *promoter:tYFP/GUS/GFP* expressing *Lotus* roots can be found in the indicated publications. n.d.: not detected.

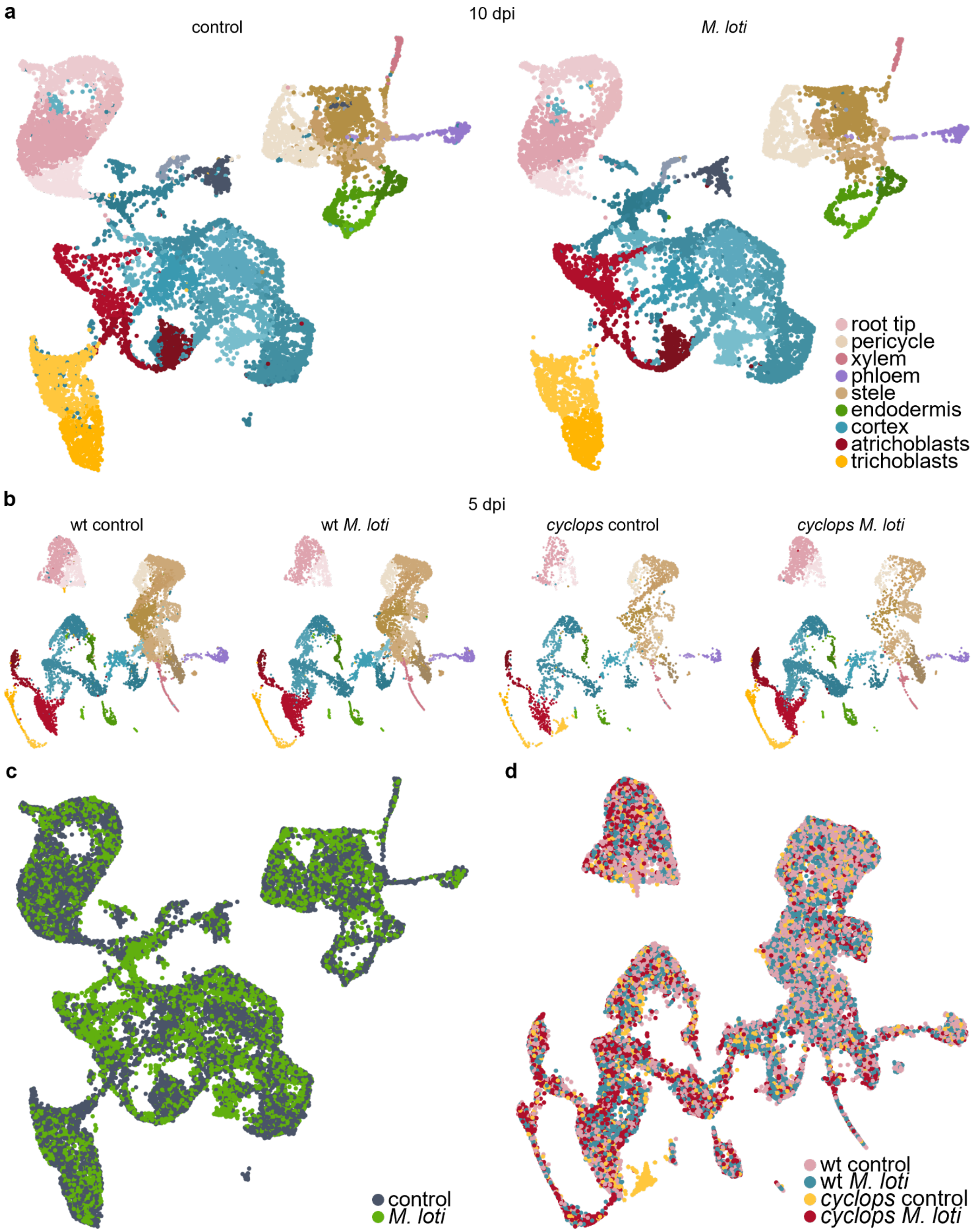

Supplementary figure 4. UMAPs split by treatment. (a) UMAP of the ten dpi dataset split by treatment. (b) UMAP of the ten dpi dataset split by treatment and genotype. (c) UMAP of the integrated ten dpi dataset colored by treatment. (d) UMAP of the integrated five dpi dataset colored by treatment and genotype.

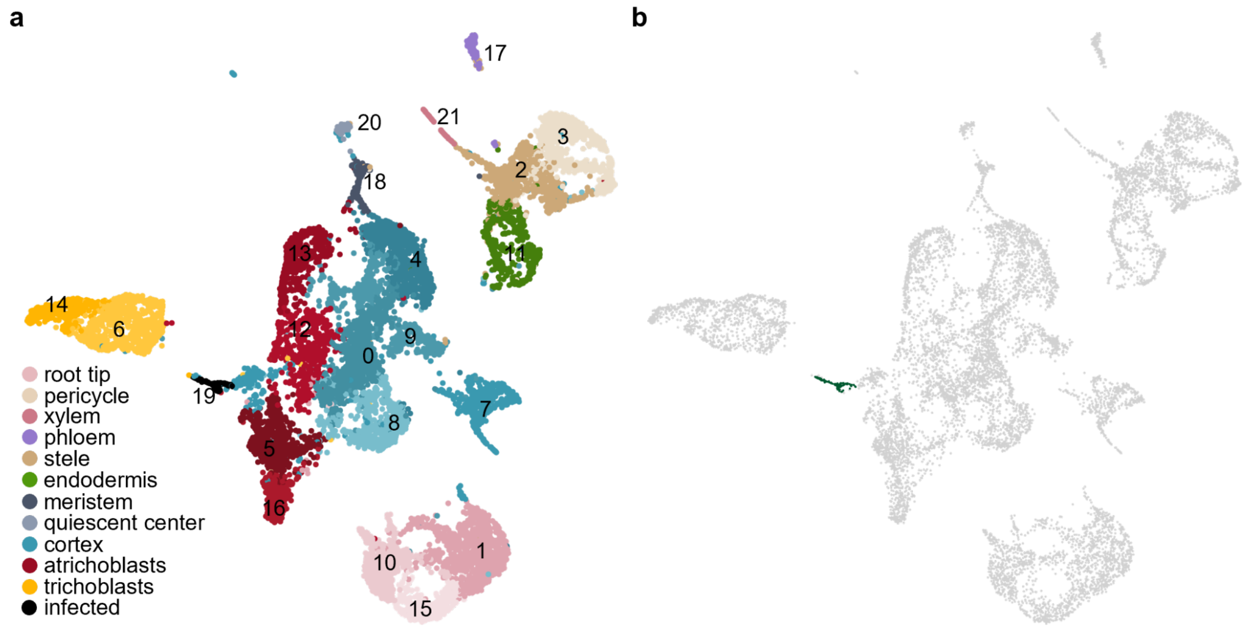

Supplementary figure 5. UMAP of *M. loti* inoculated root cells ten dpi. (a) Cluster annotations (b) Infected cells are highlighted in green.

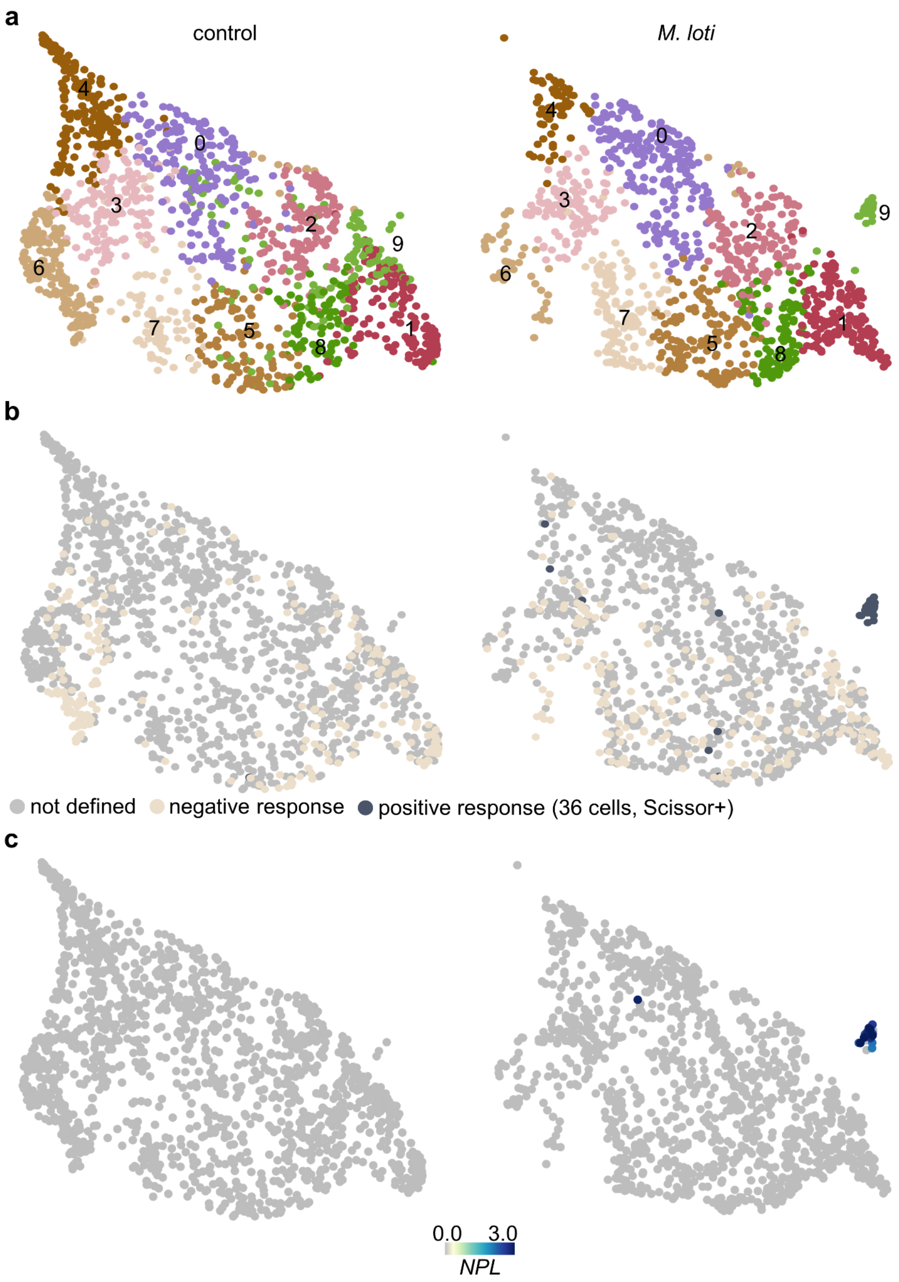

Supplementary figure 6. Reclustering of root hair cells from the 10 dpi samples. (a) UMAP of reclustered control and *M. loti* infected root hair cells ten dpi. (b) Identification of infected root hair cells by Scissor using recently published root hair bulk RNA-seq data [38]. Positively (Scissor+, dark grey) and negatively (sand-colored) responding RH cells from control and *M.loti* inoculated samples are depicted respectively. (c) Normalized expression of the Scissor+ cell marker gene *NPL*.

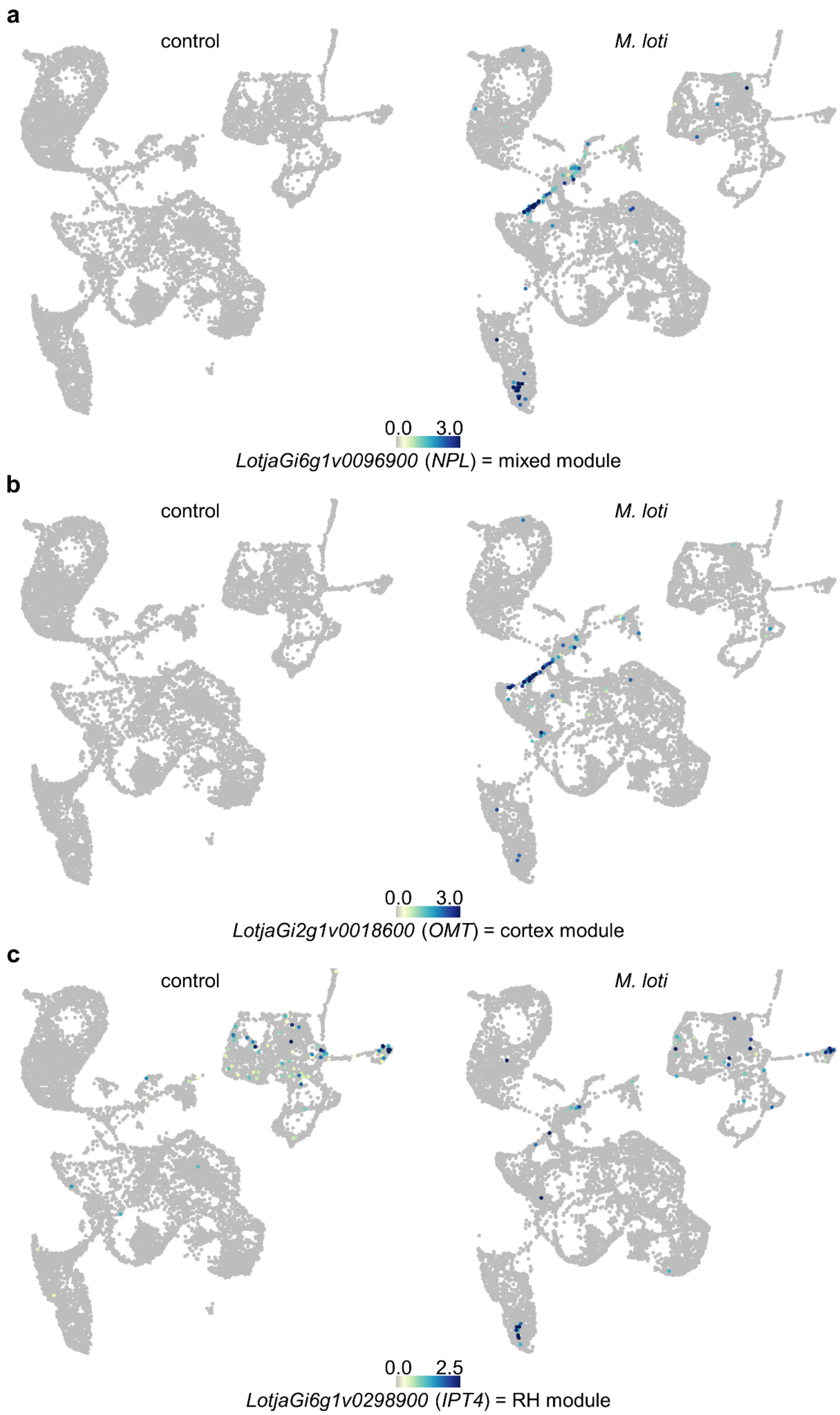

Supplementary figure 7. Markers for infected cells across root tissues. Normalized expression of mixed module marker gene *NPL* (a), the cortex module marker gene *OMT* (b) and the RH module marker gene *IPT4* (c) in control and *M. loti* inoculated samples ten dpi.

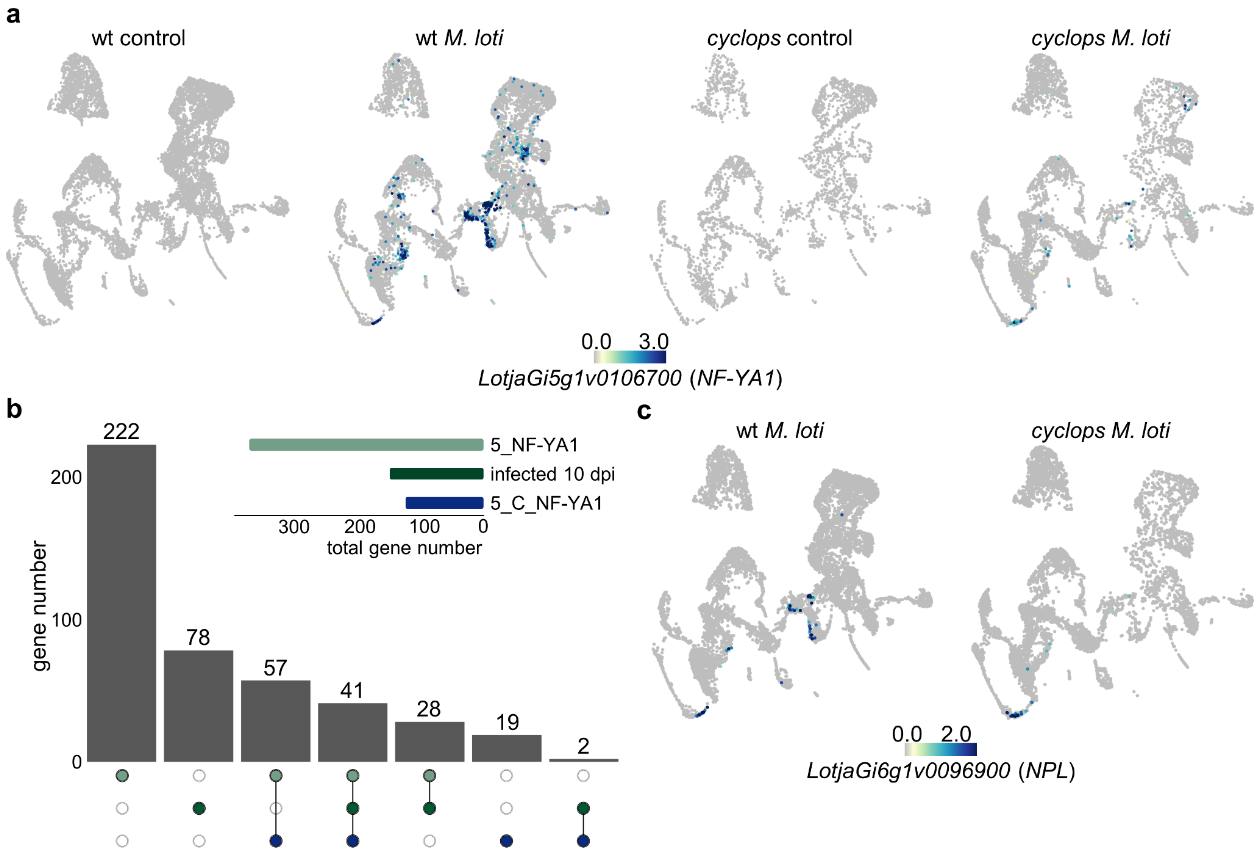

Supplementary figure 8. Comparison of *NF-YA1* expressing cells at 5 dpi with 10 dpi infected cells. (a) Normalized expression of *NF-YA1* in control and *M. loti* inoculated wild-type and *cyclops* root cells five dpi. (b) Upset plot comparing gene lists of RH and cortical cells expressing *NF-YA1* (5_NF-YA1) with infected cells ten dpi (infected ten dpi) and cortical cells expressing *NF-YA1* (5_C_NF-YA1). (c) Normalized expression of *NPL* in *M. loti* inoculated wild-type and *cyclops* root cells five dpi.

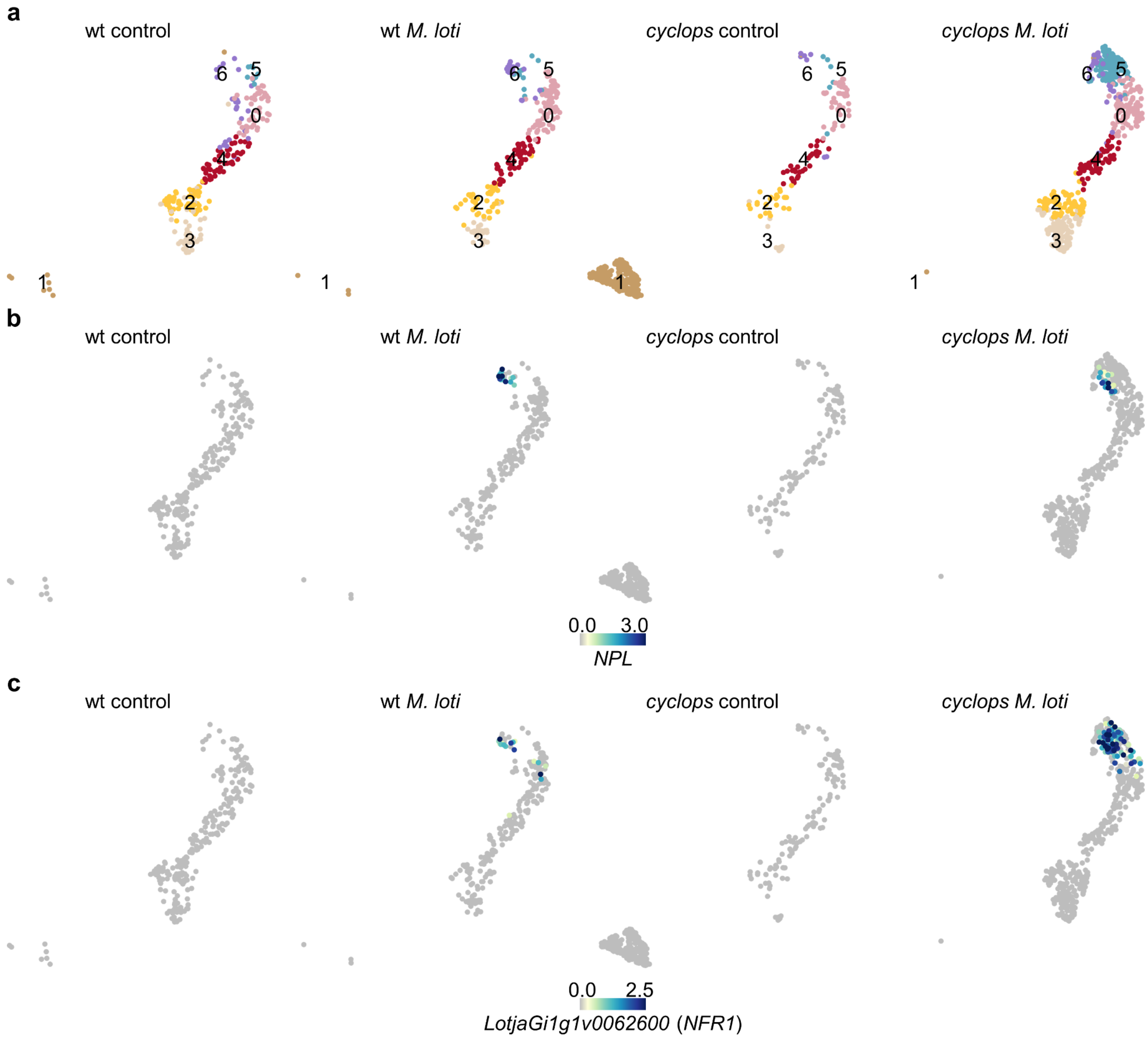

Supplementary figure 9. UMAP of reclustered control and *M. loti* infected wild-type and cyclops root hair cells five dpi. (a) Cluster annotations. Normalized expression of *NPL* (b) and *NFR1* (c) in control and *M. loti* inoculated wild-type and *cyclops* root cells five dpi.

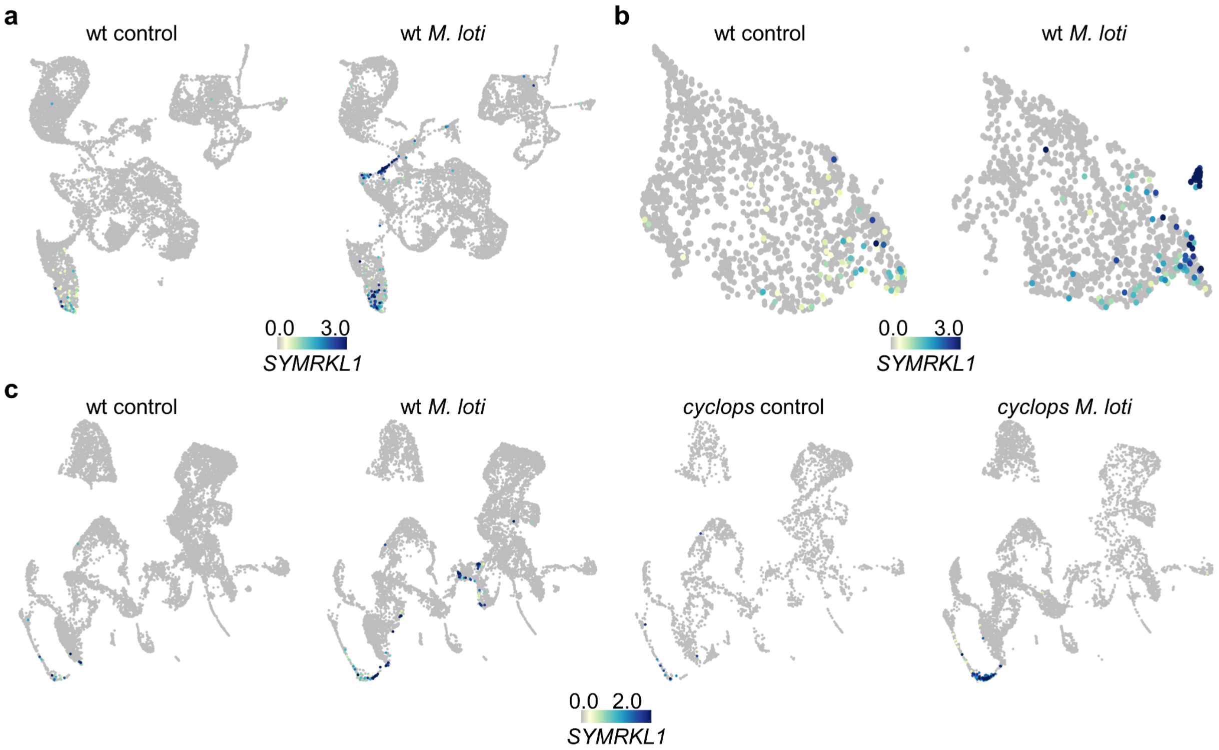

Supplementary figure 10. *SYMRKL1* expression. (a) Normalized expression of *SYMRKL1* in all root cells (a) and root hair cells (b) of control and *M. loti* inoculated samples at 10 dpi. (c) Normalized expression of *SYMRKL1* in control and *M. loti* inoculated wild-type and *cyclops* root cells 5 dpi.

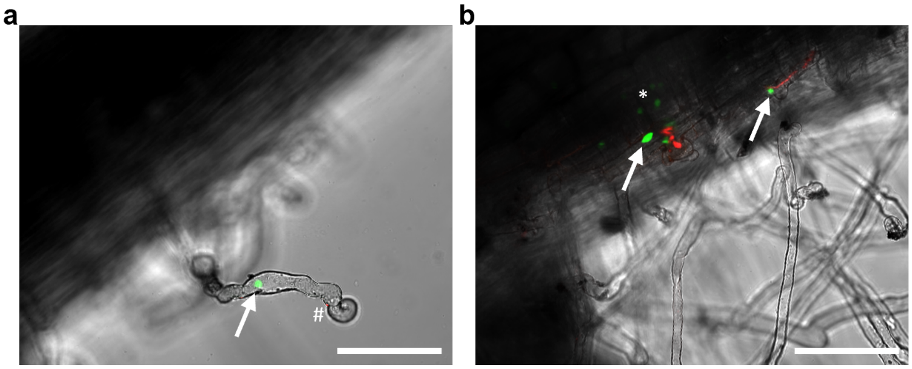

Supplementary figure 11. Colocalization of *SYMRKL1* expression with rhizobium infection. Images of *SYMRKL1:tYFPnls* expressing *Lotus* roots 10 dpi with dsRed expressing R7A a) during rhizobia attachment and b) during IT formation. Arrows indicate root hairs and the star cortical cells with green fluorescence. # indicates first rhizobial attachment. Note that the root hair is curled in response to the rhizobia and that the red dot representing the rhizobia is very small, but is visible at 200% magnification. Scale bar: 100 μm.

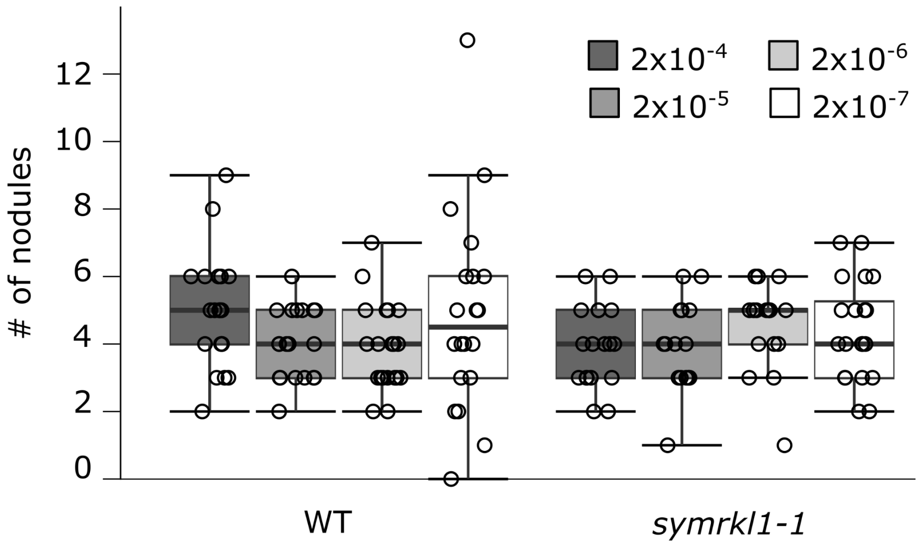

Supplementary figure 12. Nodule number of wild-type and *symrkl1* plants 21 dpi with different R7A concentrations (OD600). Statistical tests did not indicate any significance (two-way ANOVA; p ≤ 0.05; n(WT, 2x10-4) = 20, n(WT, 2x10-5) = 19, n(WT, 2x10-6) = 20, n(WT, 2x10-7) = 20, n(*symrkl1-1*, 2x10-4) = 18, n(*symrkl1-1*, 2x10-5) = 16, n(*symrkl1-1*, 2x10-6) = 17, n(*symrkl1-1-*, 2x10-7) = 20). Box plot shows min, q1, median, q3 and maximum with outliers greater than 1.5x interquartile range shown individually. Exact p-values can be found in **Source Data "Supplementary figure 12 raw and statistics".** Experiment has been successfully replicated twice.

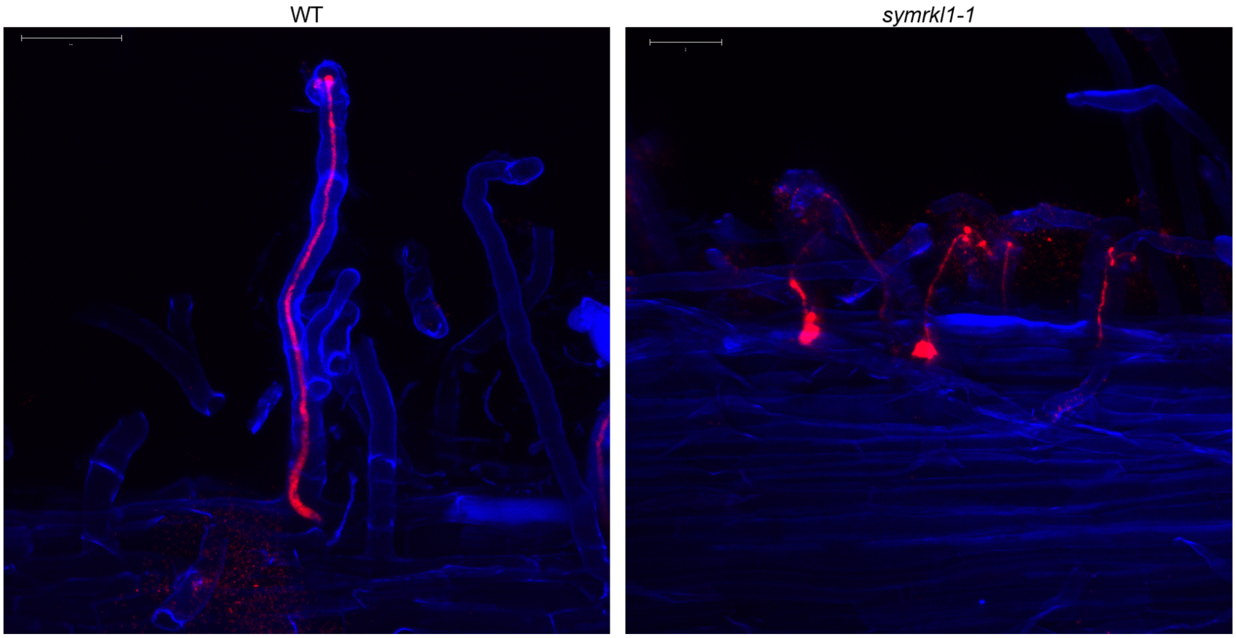

Supplementary figure 13. Original pictures from Figure 5f. Scale bar: 50 µm.
