# Supplementary material for: Single-cell analysis identifies genes facilitating rhizobium infection in Lotus japonicus
Source: Nat Commun. 2023 Nov 7;14:7171. doi: 10.1038/s41467-023-42911-1 (PMC10630511; doi:10.1038/s41467-023-42911-1)
Supplement: Supplementary file 3 — Reporting Summary [file 41467_2023_42911_MOESM3_ESM.pdf]

nature portfolio

Corresponding author(s): Stig U. Andersen, Dugald Reid

Last updated by author(s): Oct 9, 2023

Reporting Summary

Nature Portfolio wishes to improve the reproducibility of the work that we publish. This form provides structure for consistency and transparency in reporting. For further information on Nature Portfolio policies, see our Editorial Policies and the Editorial Policy Checklist.

Statistics

For all statistical analyses, confirm that the following items are present in the figure legend, table legend, main text, or Methods section.

n/a	Confirmed	
☐	☒	The exact sample size (*n*) for each experimental group/condition, given as a discrete number and unit of measurement
☐	☒	A statement on whether measurements were taken from distinct samples or whether the same sample was measured repeatedly
☐	☒	The statistical test(s) used AND whether they are one- or two-sided<br>*Only common tests should be described solely by name; describe more complex techniques in the Methods section.*
☒	☐	A description of all covariates tested
☐	☒	A description of any assumptions or corrections, such as tests of normality and adjustment for multiple comparisons
☐	☒	A full description of the statistical parameters including central tendency (e.g. means) or other basic estimates (e.g. regression coefficient) AND variation (e.g. standard deviation) or associated estimates of uncertainty (e.g. confidence intervals)
☐	☒	For null hypothesis testing, the test statistic (e.g. *F*, *t*, *r*) with confidence intervals, effect sizes, degrees of freedom and *P* value noted<br>*Give P values as exact values whenever suitable.*
☒	☐	For Bayesian analysis, information on the choice of priors and Markov chain Monte Carlo settings
☒	☐	For hierarchical and complex designs, identification of the appropriate level for tests and full reporting of outcomes
☒	☐	Estimates of effect sizes (e.g. Cohen's *d*, Pearson's *r*), indicating how they were calculated

Our web collection on statistics for biologists contains articles on many of the points above.

Software and code

Policy information about availability of computer code

Data collection	no software was used for data collection
Data analysis	Excel 2016, SAS®Studio,Cell Ranger v6.1.2 ,Seurat 4.0.5, R version 4.2.1

For manuscripts utilizing custom algorithms or software that are central to the research but not yet described in published literature, software must be made available to editors and reviewers. We strongly encourage code deposition in a community repository (e.g. GitHub). See the Nature Portfolio guidelines for submitting code & software for further information.

Data

Policy information about availability of data

All manuscripts must include a data availability statement. This statement should provide the following information, where applicable:

- Accession codes, unique identifiers, or web links for publicly available datasets
- A description of any restrictions on data availability
- For clinical datasets or third party data, please ensure that the statement adheres to our policy

Source data provided. UMAPs and gene expression data can be browsed at https://lotussinglecell.shinyapps.io/lotus_japonicus_single-cell/. Raw data is available under ENA project accession PRJEB57790. The Seurat objects generated are available at Figshare (https://doi.org/10.6084/m9.figshare.23986200.v2). The scripts used for data analyses are provided on GitHub: https://github.com/LaviFechete/Lotus_Single_Cell

Research involving human participants, their data, or biological material

Policy information about studies with human participants or human data. See also policy information about sex, gender (identity/presentation), and sexual orientation and race, ethnicity and racism.

Reporting on sex and gender	n/a
Reporting on race, ethnicity, or other socially relevant groupings	n/a
Population characteristics	n/a
Recruitment	n/a
Ethics oversight	n/a

Note that full information on the approval of the study protocol must also be provided in the manuscript.

Field-specific reporting

Please select the one below that is the best fit for your research. If you are not sure, read the appropriate sections before making your selection.

[x] Life sciences [ ] Behavioural & social sciences [ ] Ecological, evolutionary & environmental sciences

For a reference copy of the document with all sections, see nature.com/documents/nr-reporting-summary-flat.pdf

Life sciences study design

All studies must disclose on these points even when the disclosure is negative.

Sample size	Sample sizes are given in figure legends. The sample numbers were chosen according to common standards in the field.
Data exclusions	No data were excluded
Replication	scRNA-seq experiments were performed once. All other experiments were replicated successfully 2 to 3 times.
Randomization	No randomization was applied, however seedlings from different genotypes were grown under the same conditions.
Blinding	No blinding was performed

Reporting for specific materials, systems and methods

We require information from authors about some types of materials, experimental systems and methods used in many studies. Here, indicate whether each material, system or method listed is relevant to your study. If you are not sure if a list item applies to your research, read the appropriate section before selecting a response.

Materials & experimental systems

n/a	Involved in the study	
[x]	[ ]	Antibodies
[x]	[ ]	Eukaryotic cell lines
[x]	[ ]	Palaeontology and archaeology
[x]	[ ]	Animals and other organisms
[x]	[ ]	Clinical data
[x]	[ ]	Dual use research of concern
[ ]	[x]	Plants

Methods

n/a	Involved in the study	
[x]	[ ]	ChIP-seq
[x]	[ ]	Flow cytometry
[x]	[ ]	MRI-based neuroimaging

Dual use research of concern

Policy information about dual use research of concern

Hazards

Could the accidental, deliberate or reckless misuse of agents or technologies generated in the work, or the application of information presented in the manuscript, pose a threat to:

No	Yes	
☒	☐	Public health
☒	☐	National security
☒	☐	Crops and/or livestock
☒	☐	Ecosystems
☒	☐	Any other significant area

Experiments of concern

Does the work involve any of these experiments of concern:

No	Yes	
☒	☐	Demonstrate how to render a vaccine ineffective
☒	☐	Confer resistance to therapeutically useful antibiotics or antiviral agents
☒	☐	Enhance the virulence of a pathogen or render a nonpathogen virulent
☒	☐	Increase transmissibility of a pathogen
☒	☐	Alter the host range of a pathogen
☒	☐	Enable evasion of diagnostic/detection modalities
☒	☐	Enable the weaponization of a biological agent or toxin
☒	☐	Any other potentially harmful combination of experiments and agents

Plants

Seed stocks	Wild type Gifu and cyclops-2 (Yano, K. et al. PNAS 105, 20540–20545, 2008) seeds were available in-house. The LORE1 lines symrkl1-1 (30085537) and symrkl1-2 (30090169) were ordered from Lotus base (http://lotus.au.dk).
Novel plant genotypes	LORE1 lines symrkl1-1 (30085537) and symrkl1-2 (30090169) were produced via LTR element transposition as described in Małolepszy et al., 2016.
Authentication	Genotypes were confirmed by PCR and were tested for nodulation and infection thread formation. No assessment of secondary effects was performed.
